# Differences in identifying healthcare associated infections using clinical vignettes and the influence of respondent characteristics: a cross-sectional survey of Australian infection prevention staff

**DOI:** 10.1186/s13756-015-0070-7

**Published:** 2015-07-19

**Authors:** Philip L. Russo, Adrian G. Barnett, Allen C. Cheng, Michael Richards, Nicholas Graves, Lisa Hall

**Affiliations:** Institute of Health and Biomedical Innovation, School of Public Health and Welfare, Queensland University of Technology, 60 Musk Ave, Kelvin Grove, QLD 4059 Australia; Griffith University, Brisbane, QLD Australia; Infectious Diseases Epidemiology Unit, Department of Epidemiology and Preventive Medicine, Monash University, Prahran, 3181 VIC Australia; Infection Prevention and Healthcare Epidemiology Unit, Alfred Health, Commercial Rd, Prahran, 3181 VIC Australia; Faculty of Medicine, Dentistry and Health, University of Melbourne, Grattan St, Parkville, 3010 VIC Australia

**Keywords:** Healthcare associated infections, Surveillance, Clinical vignettes, Data accuracy

## Abstract

**Background:**

Australia has commenced public reporting and benchmarking of healthcare associated infections (HAIs), despite not having a standardised national HAI surveillance program. Annual hospital *Staphylococcus aureus* bloodstream (SAB) infection rates are released online, with other HAIs likely to be reported in the future. Although there are known differences between hospitals in Australian HAI surveillance programs, the effect of these differences on reported HAI rates is not known.

**Objective:**

To measure the agreement in HAI identification, classification, and calculation of HAI rates, and investigate the influence of differences amongst those undertaking surveillance on these outcomes.

**Methods:**

A cross-sectional online survey exploring HAI surveillance practices was administered to infection prevention nurses who undertake HAI surveillance. Seven clinical vignettes describing HAI scenarios were included to measure agreement in HAI identification, classification, and calculation of HAI rates. Data on characteristics of respondents was also collected. Three of the vignettes were related to surgical site infection and four to bloodstream infection. Agreement levels for each of the vignettes were calculated. Using the Australian SAB definition, and the National Health and Safety Network definitions for other HAIs, we looked for an association between the proportion of correct answers and the respondents’ characteristics.

**Results:**

Ninety-two infection prevention nurses responded to the vignettes. One vignette demonstrated 100 % agreement from responders, whilst agreement for the other vignettes varied from 53 to 75 %. Working in a hospital with more than 400 beds, working in a team, and State or Territory was associated with a correct response for two of the vignettes. Those trained in surveillance were more commonly associated with a correct response, whilst those working part-time were less likely to respond correctly.

**Conclusion:**

These findings reveal the need for further HAI surveillance support for those working part-time and in smaller facilities. It also confirms the need to improve uniformity of HAI surveillance across Australian hospitals, and raises questions on the validity of the current comparing of national HAI SAB rates.

## Introduction

Despite the absence of a standardised national healthcare associated infection (HAI) surveillance program in Australia, public reporting of HAI rates has commenced. Annual hospital level HAI *Staphylococcus aureus* bloodstream (SAB) infection rates have been reported publicly since 2012–13 [1]. Although national safety and quality health service standards mandate HAI surveillance [2], there is a large variation in HAI surveillance processes across Australia’s eight States and Territories [3, 4]. Although a national definition for SAB does exist [5], a major difference is the varying use of the National Health and Safety Network (NHSN) definitions [6] with or without local modifications to identify other HAIs [4]. It is unclear how much this variation influences the interpretation and application of definitions and subsequent HAI rates.

Whilst benchmarking and public reporting of HAI is new to Australia, it has been common in several countries for some time, including the USA, England, and France [7]. Nevertheless, there remains significant concern regarding the use of HAI data as performance indicators, particularly in light of insufficient standardisation of events being monitored [8, 9].

If HAI rates are used as quality indicators, data must be robust and reliable [10]. A recent study by Keller et al. identified low inter-rater reliability between those performing HAI surveillance and concluded that such discordance could “dramatically affect not only hospital reputations but also hospital reimbursement” [11]. Despite the lack of evidence demonstrating a reduction of HAI rates using financial incentives [12, 13], one Australian State has recently implemented financial penalties for preventable HAI bloodstream infections [14].

If Australia is to commence public reporting of other HAI data, it is important to be assured the data is robust and reliable. The objective of this study was to measure agreement in HAI identification, classification, and calculation of HAI rates amongst those undertaking HAI surveillance in Australian hospitals using a series of clinical vignettes. We also investigated if differences amongst those undertaking surveillance influenced their responses.

## Method

### Study instrument

A total of seven vignettes representing HAI surveillance situations that may occur in the acute care setting were developed as part of a larger cross-sectional survey which explored HAI surveillance practices in Australian hospitals [4]. The vignettes were based on those published in similar studies and in a local implementation guide [11, 15, 16], and were further developed in collaboration with infection prevention experts from a jurisdictional surveillance program. As not all hospitals undertake surveillance on the same type of infection, the survey was designed so that participants only answered those vignettes on which they undertook surveillance. For example, if a respondent indicated they did not perform surveillance on central line associated bloodstream infections (CLABSI), they were not presented with a vignette describing a potential CLABSI.

The vignettes were categorised into either a surgical site infection (SSI) or bloodstream infection. These types of infection were included as they represent the most common types of HAI surveillance undertaken. The first was specific to those undertaking SSI surveillance on coronary artery bypass graft surgery (CABG) to identify how they calculated an infection rate if more than one wound site was involved. A gastrointestinal surgery vignette was designed to be a straightforward case and therefore considered a positive control. The other SSI vignette was slightly more challenging in that it sought clarification as to whether or not the SSI was an organ space or deep SSI.

The SAB vignette asked respondents to indicate if they would classify it as healthcare associated. Three central line associated bloodstream infection (CLABSI) vignettes sought to identify differences regarding local modifications of the NHSN definitions, and the application of either 48 h or 2 calendar days as the marker of hospital acquisition.

For each vignette, participants were instructed to answer applying their “usual definitions and methods”.

The survey was constructed using a secure online tool and piloted by four current and two former infection prevention staff. The pilot participants provided feedback on clarity, simplicity, flow and logic of the survey. After minor amendments, the survey was further piloted by two of the six involved in the initial pilot.

### Population and recruitment

The survey was administered to infection prevention nurses who undertake HAI surveillance from both public (government funded) and private acute care facilities with more than 50 beds. This size facility was targeted as they were considered more likely to undertake HAI surveillance on a routine basis.

Recruitment was through an open invitation email distributed through the Australasian College of Infection Prevention and Control (ACIPC) list server. Coordinators of State and Territory surveillance programs, where they existed, were contacted and requested to encourage those in their State and Territory to complete the survey. Members of the Australian Commission on Safety and Quality in Health Care HAI Advisory Committee were requested to overtly support completion of the survey to their peers and colleagues. The email requested all recipients to forward on to others who may not have received it.

No identifying details of participants or their facilities were requested. Ethics permission was granted by the University Human Research Ethics Committee, Queensland University of Technology (1400000339).

### Statistical analysis

Agreement for the SSI and CLABSI vignettes was calculated as the proportion of responses considered correct using NHSN definitions [6], and for the SAB vignette according to the Australian SAB definition [5]. Data was analysed using Stata, version 13 (Stata Corp, College Station, Texas).

#### Single variable predictors of correct answers

For each vignette, univariate analysis using logistic regression was used to generate an odds ratio of answering correct depending on the participants’ characteristics. To examine all vignettes combined, a Poisson regression was used to analyse the total number correct across all vignettes, with an adjustment to the denominator, as participants only answered those vignettes on which they undertook surveillance. The results are presented as risk ratios and 95 % confidence intervals, where a risk ratio above 1 means a greater ‘risk’ of a correct answer. To make these results comparable with the logistic regression model using individual vignettes, the odds ratios from the logistic regressions were converted to risk ratios [17].

To explore the influence of the location (i.e. State or Territory of respondent), a Kruskall–Wallis test was used for each individual vignette and the combined analysis of the total number correct.

#### Multivariable predictors of correct answers

In an attempt to identify independent predictors of answering correct, a multivariable Poisson model of the total number correct was developed from characteristics identified in the Poisson univariate analysis that had a *p*-value under 0.5. A high *p*-value threshold was used to ensure that all potentially important variables were considered. To check for multicollinearity, the variance inflation factor (VIF) of each variable was explored. Variables with a VIF of 5 or above indicating high collinearity were removed from final multivariable model.

## Results

A total of 92 responses to the vignettes were received. All respondents were registered nurses with an average age of 49 and a mean of 12 years of experience working in infection prevention. There was representation from each of the eight States and Territories in Australia. The majority of respondents worked as part of a team (73 %) and in public facilities (80 %). Only 51 % reported having been trained in HAI surveillance. The median number of vignettes answered was 5 out of a maximum of 7 (Table 1).

**Table 1:** Number of vignettes answered by respondents

Number of vignettes completed	Percentage of 104^a^ participants completing
0	12 %
1	6 %
2	4 %
3	21 %
4	8 %
5	2 %
6	31 %
7 (maximum)	17 %

^a^104 responses represent all those who completed the online survey, 12 did not complete any vignettes

A summary of each vignette, response options and response rates are listed in Table 2. The number of respondents varied from 23 for Vignette 1–85 for Vignette 5. The control vignette was correctly answered by all respondents, however the correct response rates for the other vignettes varied from 53 to 75 % (Table 2).

**Table 2:** Summary of vignettes and responses (responses in bold indicate correct response)

Vignette summary (*n* = responses)	Response options	Response rate (95 % CI)
1) CABGS patient with 2 SSI and 3 incisions (*n* = 23)	1 SSI from 1 procedure	17 % (5–39 %)
	**2 SSI from 1 procedure**	**74 % (52–90 %)**
	2 SSI from 3 procedures	9 % (1–28 %)
2) Straightforward SSI following hip replacement (*n* = 81)	**Yes SSI**	**100 % (96–100 %)** ^a^
	No SSI	0 %
3) SSI following bowel resection with collection requiring surgical drainage (*n* = 81)	**Organ space SSI**	**72 % (60–81 %)**
	Deep SSI	28 % (19–40 %)
4) Presentation with infected leg ulcer with subsequent SAB during admission (*n* = 84)	**Yes HAI SAB**	**53 % (42–64 %)**
	No HAI SAB	47 % (36–58 %)
5) CLABSI if applying pre 2008 NHSN criteria 2b (*n* = 57)	Yes CLABSI	25 % (14–38 %)
	**No CLABSI**	**75 % (62–86 %)**
6) ICU attributable CLABSI (*n* = 56)	**Yes CLABSI**	**63 % (49–75 %)**
	No CLABSI	38 % (25–51 %)
7) CLABSI if using 2 calendar days but not 48 h (*n* = 55)	**Yes CLABSI**	**60 % (46–73 %)**
	No CLABSI	40 % (27–54 %)

*95 % CI* 95 % Confidence Intervals, *CABGS* Coronary artery bypass surgery, *SSI* Surgical site infection, *HAI* Healthcare associated infection, *SAB Staphylococcus aureu*s bloostream bacteraemia, *CLABSI* Central line associated bloodstream infection

^a^Exact 95 % confidence interval

### Predictors of correct answers

Univariate analysis identified three factors that were statistically significantly associated with the outcome of two of the vignettes (Table 3). For Vignette 3, which challenged the responder with the difference between classifying a SSI as either an organ space infection or a deep infection, those who worked in a team were more than twice as likely to respond correctly (RR = 2.16, [95 % CI: 1.14, 2.97]) The State or Territory of the respondents was also statistically significantly associated with a correct answer (*p* = 0.045, Kruskall–Wallis test).

**Table 3:** Univariate logistic regression analysis of vignette and respondent characterstics, with the Kruskall–Wallis test of influence of State or Territory

Variable (proportion of respondents)	Vignette 1	Vignette 3	Vignette 4	Vignette 5	Vignette 6	Vignette 7
	RR	RR	RR	RR	RR	RR
*n* = 92	(95 % CI)	(95 % CI)	(95 % CI)	(95 % CI)	(95 % CI)	(95 % CI)
Hospital over 200 beds (64 %)	n/a	1.15 (0.47, 2.10)	1.00 (0.58, 1.36)	0.94 (0.30, 2.15)	0.56 (0.14, 1.41)	1.13 (0.44, 1.90)
Hospital over 400 beds (38 %)	0.95 (0.11, 3.07)	1.50 (0.71, 2.42)	1.10 (0.72, 1.41)	2.42** (1.09, 3.45)	1.02 (0.46, 1.74)	1.07 (0.51, 1.72)
Academic degree or higher (72 %)	0.95 (0.01, 3.24)	1.41 (0.58, 2.41)	1.33 (0.91, 1.59)	1.02 (0.33, 2.27)	0.56 (0.14, 1.41)	1.36 (0.59, 2.05)
Public hospital (79 %)	1.40 (0.14, 3.30)	0.97 (0.29, 2.04)	0.76 (0.32, 1.25)	1.27 (0.37, 2.72)	1.74 (0.71, 2.46)	1.31 (0.44, 2.12)
Less than 5 years infection control experience (23 %)	1.07 (0.92, 3.15)	0.50 (0.16, 1.35)	0.66 (0.27, 1.13)	1.02 (0.19, 2.53)	0.63 (0.18, 1.56)	1.86 (0.85, 2.42)
Formal surveillance training (48 %)	1.76 (0.29, 3.44)	1.23 (0.54, 2.20)	0.70 (0.35, 1.11)	1.02 (0.19, 2.53)	1.25 (0.54, 2.02)	1.22 (0.56, 1.91)
Trained by central organisation (21 %)	1.07 (0.20, 2.82)	1.53 (0.58, 2.66)	1.02 (0.52, 1.44)	2.27 (0.53, 3.68)	1.04 (0.37, 1.92)	1.00 (0.35, 1.82)
Surveillance skills assessed (17 %)	n/a	0.99 (0.32, 2.34)	0.72 (0.27, 1.25)	0.32*** (0.09, 0.98)	1.94 (0.85, 2.60)	1.05 (0.37, 1.92)
Work in a team (73 %)	2.04 (0.04, 3.81)	2.16* (1.14, 2.97)	1.02 (0.58, 1.40)	1.02 (0.33, 2.27)	0.85 (0.26, 1.75)	1.69 (0.86, 2.24)
Daily access to Infectious Diseases Physician (59 %)	1.73 (0.18, 3.49)	0.89 (0.35, 1.77)	1.05 (0.64, 1.39)	0.53 (0.14, 1.55)	0.58 (0.16, 1.38)	1.17 (0.48, 1.90)
Daily access to Epidemiologist (1 %)	1.35 (0.14, 3.73)	1.14 (0.25, 2.99)	1.39 (0.68, 1.71)	1.45 (0.23, 3.37)	1.63 (0.32, 2.67)	1.20 (0.27, 2.29)
Daily access to Microbiologist (64 %)	1.73 (0.18, 3.49)	0.90 (0.34, 1.81)	0.82 (0.44, 1.23)	1.39 (0.51, 2.65)	1.00 (0.35, 1.87)	0.91 (0.31, 1.72)
Effective full time staff >3 (27 %)	0.49 (0.05, 2.34)	0.84 (0.23, 2.11)	0.69 (0.29, 1.19)	0.76 (0.24, 1.82)	0.95 (0.30, 1.90)	0.39 (0.08, 1.19)
Rarely or never have access to an ICP with more experience (43 %)	0.49 (0.08, 1.91)	1.51 (0.69, 2.49)	1.07 (0.66, 1.41)	0.66 (0.23, 1.60)	0.93 (0.36, 1.74)	1.04 (0.43, 1.78)
Work part time (34 %)	0.26 (0.04, 1.40)	0.57 (0.21, 1.34)	0.83 (0.44, 1.25)	0.72 (0.21, 1.86)	0.63 (0.18, 1.56)	1.05 (2.46, 1.92)
Kruskall–Wallis test for State/Territory (*P*-value)	0.0875	0.0454	0.4163	0.0427	0.2826	0.3389

*RR* Risk Ratio, *95 % CI* 95 % Confidence Interval

*p = 0.011 **p = 0.033 ***p = 0.049

Vignette 5 explored the difference between the current NHSN criteria for CLABSI against 2008 criteria. Working in a hospital with over 400 beds more than doubled the likelihood of a correct answer (RR = 2.42, [95 % CI: 1.09, 3.45]), but those who have had surveillance skills assessed were less likely to have a correct answer (RR = 0.32, [95 % CI: 0.09, 0.98]). There was evidence that the proportion answering correctly varied between State or Territory (Kruskal–Wallis test: *p* = 0.043).

Those characteristics that were more frequently associated with a correct response across all vignettes were: working in a hospital over 400 beds, having been formally trained in surveillance, being trained by a central organisation, working in a team, and having daily access to an epidemiologist. The characteristic most commonly associated with an incorrect response was working part-time.

No statistically significant factors were identified for the total number correct, but characteristics most strongly associated with a correct response were working in a team RR = 1.15 (95 % CI: 0.89, 1.49) and daily access to an epidemiologist RR = 1.15 (95 % CI: 0.81, 1.62). Working part-time was most strongly associated with an incorrect answer RR = 0.89 (95 % CI: 0.69, 1.14).

### Multivariable analysis

Two multivariable models were developed (Table 4). Characteristics from the univariate analysis that had a *p*-value < 0.5 were included in the first model (Model A). The variable “Work in a Team” was found to have a VIF of 5. Therefore, a second multivariate model (Model B) was generated following the omission of “Work in a Team”.

**Table 4:** Multivariable analysis of respondent characterstics using poisson regression of the number of correct answers

	Model A - includes “Work in a team”		Model B - excludes “Work in a team”	
Variable	Risk ratio (95 % CI)	*P* value	Risk ratio (95 % CI)	*P* value
Hospital over 400 beds	0.99 (0.76, 1.31)	0.963	1.04 (0.81, 1.32)	0.766
Academic degree or higher	1.08 (0.83, 1.39)	0.583	1.08 (0.84, 1.40)	0.545
Less than 5 years infection control experience	0.96 (0.71, 1.31)	0.808	0.96 (0.71, 1.30)	0.806
Daily access to Epidemiologist	1.12 (0.78, 1.61)	0.548	1.12 (0.77, 1.61)	0.555
Work part time	0.92 (0.69, 1.22)	0.555	0.91 (0.69, 1.20)	0.503
Work in a team	1.11 (0.82, 1.50)	0.509	-	-

*95 % CI* 95 % Confidence Interval

A risk ratio above 1 indicates an increased chance of a correct answer

For both models, the probability of getting a correct answer increased by 12 % if the respondent had daily access to an epidemiologist, and 8 % if they had an academic degree or higher. For Model A the probability increased by 11 % if they worked as part of a team. Both models also identified that incorrect answers were more common for respondents who were part-time or with less than five years experience. No statistically significant factors were identified.

## Discussion

This study has identified disparity in HAI identification, classification, and calculation of HAI rates using clinical vignettes in large acute care Australian hospitals. Although one vignette returned an encouraging result of 100 % correct response rate, it was included as a positive control. The range of responses of 53–75 % for the other six vignettes follows on from recent findings describing the broad variation amongst surveillance practices in Australia [4], and infer that comparison between hospitals, States and Territories, and any aggregation of existing data will be flawed. This is implicit from the following findings.

First, aggregation of SSI rates following CABGs will result in an underestimation of the true rate whilst some hospitals, States and Territories persist in using each incision as the denominator to calculate a rate. Second, the inability to distinguish between organ space and deep space means that any aggregated SSI data reported by type of infection will likely be unreliable and incomparable. Third, the present use of both 48 h or 2 calendar days as criteria for CLABSI acquisition clearly affects the CLABSI rate reported. Fourth, even though a national definition for SAB exists (unlike the potential HAIs described in other vignettes) when presented with a complex SAB event the ability to correctly identify it is moderate. This is important as current SAB rates, that are publicly reported on a safety and quality website in Australia encouraging hospital comparisons [1], could be misleading.

The univariate analysis findings suggest that those from larger hospitals and in States with established programs are more likely to be in agreement with current NHSN HAI definitions. This could be explained by the team environment of larger hospitals which may provide improved knowledge from greater learning opportunities, and the training provided by the established programs.

Although no statistically significant predictors were identified in the multivariable analysis, the results from both models indicate that those with less experience and those who work part-time require increased support and training to identify HAIs.

Daily access to an epidemiologist was positively associated with a correct answer for all vignettes and also both models of the multivariable analysis. Given that only 1 % of respondents have daily access to an epidemiologist, this may be a proxy for other factors (e.g., a thriving research culture) that have not been identified in this study and is worthy of further exploration.

The results of this study are consistent with recent international studies that have identified broad variation in the identification of both SSI and CLABSI within and between HCW groups [11, 15, 18–21]. Similar to Keller’s study [11], we attempted to identify characteristics that may act as independent predictors of a correct response. Keller identified that those with a clinical background were more likely to identify a HAI correctly. All the respondents to this study were infection prevention nurses with a clinical background and like Keller, no other significant predictors were identified in a multivariable model.

Unlike a recent study using clinical vignettes, [22] we were unable to estimate sensitivity and specificity for this study. Although most hospitals use HAI definitions based on NHSN, there is no uniform national definition for surgical site infection or CLABSI in Australia, and so there is no gold standard available to measure sensitivity and specificity. Also, the emphasis and main objective of this study was to measure agreement, rather than sensitivity and specificity amongst participants.

There are limitations to this study. Selection bias and small numbers may influence the results. Despite the small number of responses, variation in agreement is clearly evident. A survey response rate was unable to be calculated as the number of infection prevention staff in Australia is unknown [23], and we are uncertain how many received the survey. Approximately 500 ACIPC members subscribe to the list server, (personal communication, ACIPC secretary June 2014), but not all undertake HAI surveillance, nor are all infection prevention staff members of ACIPC. It is estimated there are approximately 215 acute public hospitals with more than 50 beds in Australia [24], and our respondents were from all States and Territories with a broad range of experience working in different sized hospitals, and so we are confident this is representative of those undertaking HAI surveillance. Not all participants answered each vignette, as they were only required to answer vignettes relevant to the type of surveillance they usually perform, therefore some vignettes were correctly not answered. Completing vignettes online does not represent reality, and many infection prevention staff will discuss potential HAIs before making a decision, particularly those who work in teams.

A major strength of this study is its anonymity in that there was no pressure influencing the respondents if they had any uncertainty. This in fact may represent a more accurate reflection of infection prevention staff true understanding.

## Conclusion

The results of this study have been derived from those who are currently charged with collecting HAI data, and indicate that training and support resources for those in smaller facilities who work part-time needs to be strengthened.

Before national reporting can be established, robust standardised surveillance processes need to be implemented. Presently, the validity of existing SAB data is questionable, and the temptation to aggregate any existing HAI rates to generate national data must be avoided.

## Abbreviations

HAI: Healthcare associated infection
SAB: *Staphylococcus aureus* bacteraemia
CLABSI: Central line associated bloodstream infection
CSSI: Surgical site infection
CABG: Coronary artery bypass graft
NHSN: National Health and Safety Network
ACIPC: Australasian College for Infection Prevention and Control
VIF: Variance inflation factor
RR: Risk ratio
CI: Confidence Interval
